# A functionally neutral single chain antibody to measure beta‐1 integrin uptake and recycling

**DOI:** 10.1111/tra.12754

**Published:** 2020-07-21

**Authors:** Ashley M. Lakoduk, Zuzana Kadlecova, Sandra L. Schmid

**Affiliations:** ^1^ Department of Cell Biology UT Southwestern Medical Center Dallas Texas USA; ^2^ Present address: Cambridge Institute for Medical Research Cambridge UK

**Keywords:** endocytosis, integrin, recycling, scFv, trafficking

## Abstract

Integrin‐mediated cell adhesion and signaling are critical for many physiological processes. The dynamic turnover of integrins and their associated adhesion complexes through endocytic and recycling pathways has emerged as an important mechanism for controlling cell migration and invasion in cancer. Thus, the regulation of integrin trafficking and how this may be altered by disease‐specific molecular mechanisms has generated considerable interest. However, current tools available to study integrin trafficking may cause artifacts and/or do not provide adequate kinetic information. Here, we report the generation of a functionally neutral and monovalent single chain antibody to quantitatively and qualitatively measure β1 integrin trafficking in cells. Our novel probe can be used in a variety of assays and allows for the biochemical characterization of rapid recycling of endogenous integrins. We also demonstrate its potential utility in live cell imaging, providing proof of principle to guide future integrin probe design.

## Synopsis

The dynamic turnover of integrins through endocytic trafficking pathways has emerged as a key mechanism for cell migration and invasion. Lakoduk et al. report the generation of a functionally neutral and monovalent antibody‐based probe to track and measure endogenous beta‐1 integrin uptake and fast recycling in multiple cell types. Their tool, scFv^K20^, serves as proof of principle inspiration for future integrin probe design.

## 1 INTRODUCTION

In multicellular organisms, cells must sense and interpret contextual information received through direct physical interactions with the extracellular matrix (ECM) in order to maintain proper cellular identity. The formation of integrin‐based cell‐ECM adhesion complexes is essential during development and often dysregulated in pathological conditions, including cancer. Integrins are abundant cell surface αβ heterodimeric adhesion receptors that anchor the intracellular cytoskeleton to the ECM.^1^ Integrins can adopt a bent/closed (inactive) conformation, or an extended/open (active) conformation, with possible intermediate “priming” states.^2^ Integrin conformation and activity status can be influenced by either ECM/ligand binding (“outside‐in”) or macromolecular protein complex recruitment and binding to the integrin cytoplasmic tails (“inside‐out”), allowing integrins to signal bi‐directionally across the plasma membrane.^1^ The signaling driven by these integrin conformational changes plays pivotal roles in cell survival and proliferation, and can also directly modulate the cytoskeleton and control cell motility.

Intracellular trafficking is responsible for the differential targeting and distribution of membrane‐associated proteins within the cell.^3^ The dynamic turnover of cell surface integrins through endocytic and recycling pathways has emerged as a key mechanism in regulating integrin function.^4^, ^5^ Integrins have been shown to follow several endocytic pathways, including clathrin‐^6^, ^7^, ^8^, ^9^, ^10^ and caveolin‐dependent^11^, ^12^, ^13^ endocytosis, clathrin‐independent endocytosis^14^, ^15^ and macropinocytosis.^16^ Once internalized, integrins are routed to early endosomes, in which initial sorting decisions are made that determine whether the integrins are sent for degradation or recycled back to the plasma membrane. Generally, internalized integrins are not degraded but are recycled by either a direct “fast” recycling route or indirectly through the “long loop” perinuclear recycling compartment before returning to the plasma membrane.^17^, ^18^, ^19^ Rapid integrin recycling has been shown to promote malignant phenotypes in several cancer cell lines.^19^, ^20^, ^21^ Interestingly, it has also been shown that integrins can escape from late endosomes/lysosomes and efficiently recycle back to the plasma membrane.^22^ Thus, integrin trafficking plays a critical role in controlling receptor surface levels and function.

Integrin trafficking and signaling are dictated by the overlapping linear binding and sorting motifs encoded within the cytoplasmically oriented C‐termini of both α and β integrins.^17^ Protein binding and recruitment to integrin tails directly controls integrin signaling and trafficking. In fact, several groups have shown that competition for binding to integrin cytoplasmic tails directly determines receptor fate.^23^, ^24^

Our current knowledge of integrin trafficking has been based on studies using either the exogenous expression of integrins with cytoplasmic tails fused to a fluorescent protein,^10^, ^25^, ^26^, ^27^, ^28^, ^29^ antibody‐based labeling,^30^, ^31^, ^32^ or surface biotinylation^21^, ^33^, ^34^—each of which has drawbacks. For example, the efficient incorporation of cytoplasmic domain fusions into αβ heterodimers, which is required to exit the endoplasmic reticulum (ER), requires concomitant knockdown of the endogenous subunit. Moreover, the bulky cytoplasmic fusions can induce potential trafficking and signaling artifacts by disrupting protein recruitment and binding. Bivalent antibody labeling techniques can perturb trafficking routes, kinetics, or signaling by inducing receptor aggregation and activation.^35^, ^36^ In fact, many antibodies can alter the conformation, activity, and function of integrins.^2^, ^37^, ^38^ Furthermore, active and inactive β1 integrins were recently shown to spatially segregate within adhesions^39^; therefore, the use of function‐perturbing antibodies may alter this spatial coordination and downstream signaling. To circumvent these problems, many groups have used surface biotinylation to measure uptake and recycling kinetics of endogenous integrins. However, surface biotinylation requires long labeling times at 4°C, which can perturb trafficking,^40^ and lacks the sensitivity to detect fast recycling. Moreover, surface biotinylation cannot be used to address trafficking dynamics by live‐cell microscopy.

Recently, in response to concerns about the current tools used to report integrin trafficking in cells, Huet‐Calderwood et al. published the use of recombinant β1 integrins with an “ecto‐tag” inserted within the extracellular hybrid domain.^41^ Using these ecto‐tagged β1 integrins, they report a spatial bias in integrin exocytosis. Unfortunately, as with all recombinant integrin fusions, the expression of ecto‐tagged integrins must be precisely titrated so as not to induce ER accumulation or overexpression‐related artifacts.^41^, ^42^ Therefore, the creation of more straightforward tools that could be readily applied to study endogenous integrins in any cell type would vastly increase our understanding of the intricate spatiotemporal regulation of integrin trafficking.

Here, we report the design and expression and purification of a single chain variable fragment (scFv) based on the previously characterized, non‐function perturbing anti‐β1 integrin monoclonal antibody, mAb K20.^38^, ^43^, ^44^, ^45^ The purified MBP‐scFv^K20^ is functionally neutral and monovalent, and able to specifically track and quantify endogenous β1integrin trafficking itineraries in cells, without the need for cell engineering and recombinant integrin expression. We demonstrate that our probe allows for reliable tracking of rapid β1 integrin recycling, and thus serves as proof of principle for the generation of future antibody‐based probes. The future use of a single tool to directly correlate bulk biochemical assays with spatiotemporal dynamics acquired via live‐cell microscopy will provide further mechanistic insight into the regulation of integrin trafficking.

## 2 RESULTS

### 2.1 Generation of an anti‐β1 integrin scFv

**FIGURE 1:**
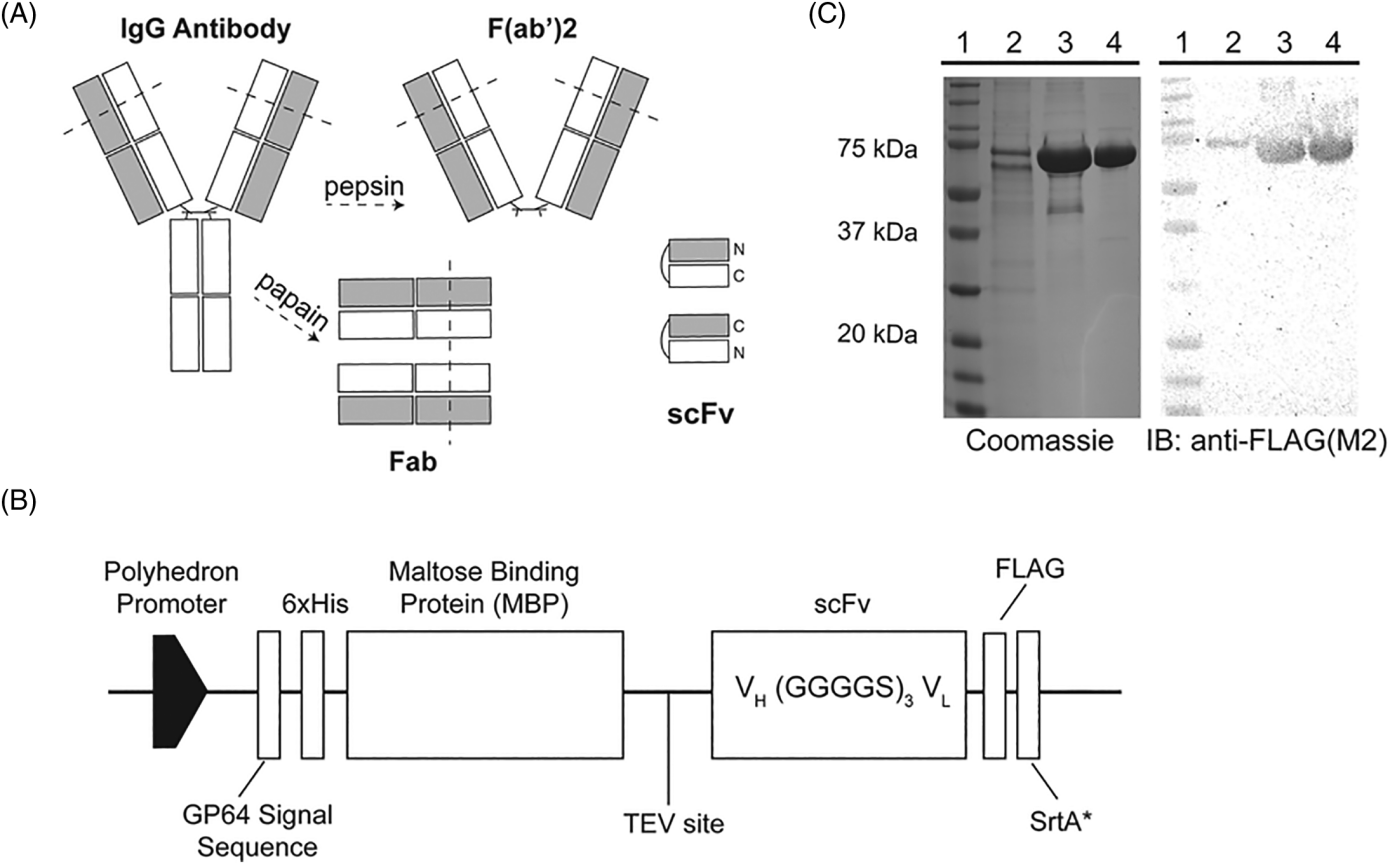
Anti‐β1 integrin single chain variable fragment design, expression, and purification. A, Schematic of IgG antibody and derivatives after pepsin and papain digestion. The ends of the IgG and Fab fragments participate in antigen binding and can be recombinantly expressed as a single‐chain variable fragment (scFv) by connecting the variable heavy (*V*_H_) and variable light (*V*_L_) domains with a flexible linker. Antibody heavy chains are in white, and light chains depicted in gray. B, Schematic of the anti‐β1 integrin scFv^K20^ construct used for baculovirus‐mediated insect cell expression under the control of a polyhedrin promoter. The cleavable GP64 signal sequence allows for protein secretion. The resultant recombinant protein is a 71 kDa N‐terminal 6x His‐tagged MBP‐fusion with a C‐terminal FLAG tag and SortaseA (SrtA*) recognition motif. MBP can be cleaved post‐purification with TEV protease. C, Representative SDS‐PAGE of harvested baculovirus‐infected insect cell supernatant (Lane 2) and subsequent purification products (Lanes 3‐4; left, Coomassie blue‐stained; right, anti‐FLAG immunoblot). Lane 1: Precision Plus protein ladder. Lane 3: pooled and concentrated IMAC affinity purification fractions. Lane 4: final purified MBP‐scFv^K20^ from peak 2, after size‐exclusion chromatography (see also Figure S1)

We sought to develop a more versatile and nonperturbing probe to quantitatively analyze endogenous β1 integrin trafficking. To this end, we generated a neutral and monovalent single chain antibody variable fragment (scFv) against β1 integrin. ScFvs represent the smallest unit of high affinity antibody‐based binding,^46^ as they contain the antigen‐binding variable heavy (*V*_H_) and variable light (*V*_L_) segments of an antibody connected by a flexible linker (Figure 1A). *V*_H_ and *V*_L_ sequences used to design the scFv were derived from mAb K20, a previously characterized, non‐function perturbing, mouse monoclonal antibody against β1 integrin.^38^, ^43^, ^44^, ^45^ The mAb K20 antibody is reported to bind the EGF repeat region in the membrane‐proximal extracellular domain of β1 integrin, and is not predicted to interfere with integrin conformation or activation.^45^ The mAb K20 sequence, which was described in a patent,^47^ was validated by mass spectrometry of commercially available antibody (Figure S1A). The resulting scFv, hereafter named scFv^K20^, contains the entire antigen binding region of the parent monoclonal antibody and therefore is expected to retain the same binding specificity.^48^

Both antibodies and scFvs require intramolecular disulfide bonds within each variable domain for correct tertiary structure. To allow for proper disulfide bond formation and protein folding through the secretory pathway,^49^ the scFv^K20^ construct was designed for secreted baculovirus‐mediated expression in insect cells. Amino acid sequences of mAb K20 were used to design a corresponding codon‐optimized cDNA, which was commercially synthesized. A schematic of the scFv^K20^ construct used for baculovirus generation is shown in Figure 1B. To increase expression and solubility, the scFv is genetically fused to a TEV‐cleavable Maltose Binding Protein (MBP).^50^, ^51^ Additionally, the construct encodes an amino‐terminal GP64 secretion signal sequence and a 6x‐Histidine (6xHis) tag for affinity purification. scFv^K20^ also contains a C‐terminal FLAG epitope for recognition by commercially‐available anti‐FLAG antibodies, and a SortaseA (SrtA*) recognition motif for in vitro site‐specific labeling.^52^ As designed, our scFv construct is versatile and can be customized for a wide range of assays.

To generate recombinant scFv^K20^ we first needed to generate recombinant baculovirus. For high‐level baculovirus‐mediated expression, the synthesized gene product was cloned into a modified pFastBacHT vector^53^ that facilitated recombinant bacmid generation using the Bac‐to‐Bac Baculovirus expression system (Thermo Fisher Scientific). Low‐titer P1 baculovirus stock was generated by transfecting validated bacmid DNA into Sf9 insect cells and harvesting supernatant after obvious signs of late‐stage viral infection (eg, signs of viral budding and cell lysis; approximately 5 days post‐transfection, Figure S1B). P1 virus stock was used to create high‐titer P2 baculovirus for use in subsequent protein expression.

For protein expression and purification, High Five insect cells were grown in serum free medium and infected with P2 baculovirus. The insect cell supernatant containing the secreted scFv^K20^ was harvested 48 hours post‐infection (Figure 1C, Lane 2). Recombinant MBP‐fused scFv^K20^ was purified by immobilized metal ion affinity chromatography (IMAC) (Figure 1C, Lane 3). Anti‐FLAG immunoblotting throughout the purification confirmed enrichment of MBP‐scFv^K20^ (Figure 1C). Following affinity purification, the MBP‐fused scFv was further enriched via size‐exclusion chromatography (SEC) to remove aggregates, which are undesirable and could induce integrin clustering and alter function (Figure 1C, Lane 4 and Figure S1C). As peak two (Figure S1C) corresponded to the expected molecular weight of MBP‐fused scFv^K20^ (ie, 71 kD) it was collected and labeled on free amines by NHS‐ (*N*‐hydroxysuccinimide ester) or SPD‐ (sulfodichlorophenol ester) conjugation with either a disulfide‐cleavable biotin or an Alexa Fluor dye, respectively, for use in subsequent biochemical and microscopy assays. The final yield was ~0.5 mg of purified MBP‐scFv^K20^ per 100 mL of harvested insect cell supernatant. If desired, TEV protease can cleave MBP from the scFv after purification. However, the MBP‐scFv^K20^ fusion retains the same binding properties as cleaved scFv^K20^ (data not shown) and MBP increases the protein's long‐term stability and solubility.^50^, ^54^ Moreover, the MBP fusion protein provides further sites for amine‐reactive labeling without perturbing scFv function. Therefore, MBP‐fused scFv was used for all subsequent assays (herein referred to as “MBP‐scFv^K20^”).

### 2.2 Monovalent MBP‐scFv^K20^ retains β1 integrin binding specificity and does not affect integrin function

**FIGURE 2:**
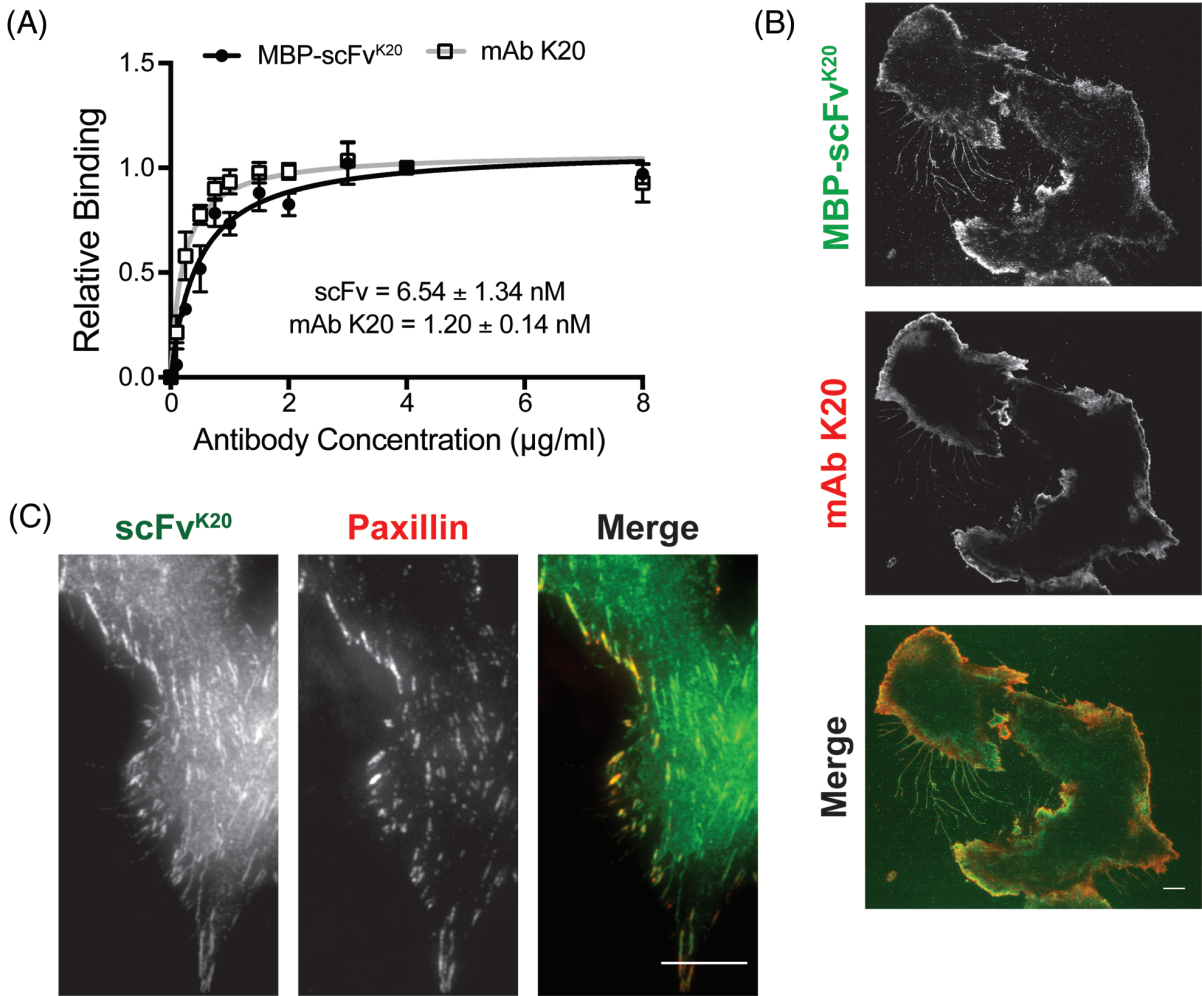
Anti‐β1 integrin scFv^K20^ retains binding specificity for β1 integrins. A, Antibody binding curves and calculated binding affinities (*K*_D_). Relative binding of antibodies was determined using a cell‐based ELISA binding assay. Values plotted are normalized to maximal binding per individual experiment. *K*_D_ was determined as the concentration of antibody at half‐maximal binding using GraphPad Prism. n = 4. Error bars represent SD. B, Representative TIR‐FM immunofluorescence images of surface β1 integrins of hTERT‐RPE 1 cells seeded on gelatin‐coated coverslips. Co‐staining of anti‐β1 MBP‐scFv^K20^ (green) and parent mAb K20 (red) antibodies. Scale bar, 10 μm. C, Representative TIR‐FM immunofluorescent images of permeabilized hTERT‐RPE 1 cells seeded on gelatin‐coated coverslips probed with media from cells secreting scFv^K20^ and anti‐FLAG secondary antibody (green) as well as the cellular adhesion marker paxillin, detected with α‐phospho‐(Y118) antibody (red). Scale bar, 10 μm

To validate that MBP‐scFv^K20^ retains its binding specificity to β1 integrin, we performed a binding isotherm using a cell‐based enzyme‐linked immunosorbent assay (ELISA). We compared MBP‐scFv^K20^ and parent mAb K20 binding to endogenous β1 integrin on the surface of H1975 cells (Figure 2A), and determined the binding affinity (*K*_D_) of MBP‐scFv^K20^ (6.54 ± 1.34 nM) to be approximately one order of magnitude less than mAb K20 (1.20 ± 0.14 nM). This decrease was to be expected, due to the loss of avidity of monovalent vs bivalent binding. Nevertheless, the *K*_D_ of MBP‐scFv^K20^ remains in the low nanomolar range (<10 nM), making it suitable for most biochemical assays.

To assess whether MBP‐scFv^K20^ and mAb K20 have the same binding specificity on cells, we compared MBP‐scFv^K20^ and mAb K20 binding to the surface of hTERT‐RPE1 cells by immunofluorescence. Immunofluorescence staining and total internal reflection fluorescence microscopy (TIR‐FM) imaging revealed significant colocalization between biotinylated MBP‐scFv^K20^ and mAb K20 (Figure 2B). The higher background signal for MBP‐scFv^K20^ likely reflects the use of a secondary Streptavidin Alexa Fluor conjugate. Further, TIR‐FM imaging analysis of permeabilized hTERT‐RPE1 cells using culture media containing secreted scFv^K20^showed its expected association with the ventral cellular membrane, as well as colocalization with the cellular adhesion marker, paxillin (Figure 2C). Taken together, we conclude that MBP‐scFv^K20^ retains similar β1 integrin binding specificity as its parent mAb K20.

**FIGURE 3:**
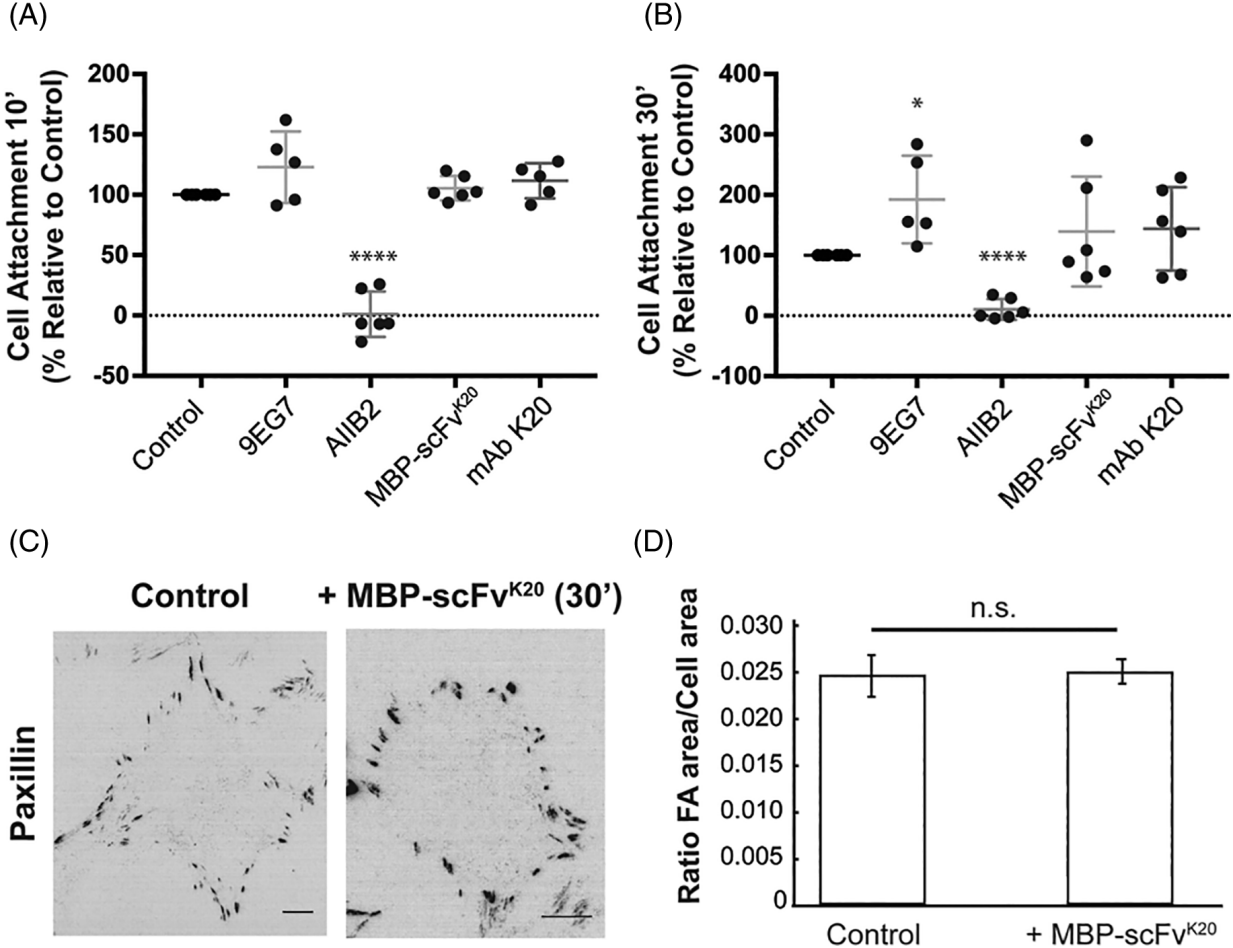
ScFv^K20^ does not perturb integrin function. A, Attachment of H1975 cells at 10 minutes or, B, 30 minutes after plating on wells coated with 0.02% gelatin and 25 μg/mL fibronectin in the absence (control) or presence of 5 μg/mL of the indicated anti‐integrin antibodies. 9EG7 is an integrin activating control, AIIB2 is an integrin inhibitory control. Cell attachment measured by crystal violet staining and the absorbance at 570 nm. Data was normalized to 100% attachment of untreated control cells. Error bars represent Mean with SD (n = 3). Unpaired t‐test was used for statistical significance. * p<0.05, **** p<0.001. C, Representative inverted TIR‐FM immunofluorescence images of H1975 cells pre‐seeded on gelatin‐ and fibronectin‐coated coverslips and incubated in the absence (control) or presence of 5 μg/mL MBP‐scFv^K20^ for 30 minutes at 37°C. Scale bar, 10 μm. D, Quantitative comparison of total detected focal adhesion area relative to the detected cell area of H1975 cells incubated as in (C), n.s., not significant. Wilcoxon Rank‐Sum non‐parametric test was used for statistical significance

As MBP‐scFv^K20^ retained β1 integrin binding specificity and colocalized with adhesion‐associated β1 integrins, we next assessed whether MBP‐scFv^K20^ perturbs integrin function. To test this, we first performed adhesion assays in H1975 cells. Cell adhesion to a matrix of gelatin and fibronectin (FN) was measured using a modified static adhesion assay that detects bound cells via crystal violet staining (see Section 4, Methods).^55^ In addition to parent mAb K20, two known β1 integrin function‐altering antibodies, the activating mAb 9EG7 and the inhibitory mAb AIIB2 were used as controls.^38^ As expected, incubation with β1 integrin activating antibody, 9EG7, increased cell attachment after 10 minutes, while the inhibitory β1 integrin antibody, AIIB2, significantly reduced cell adhesion to the gelatin‐ and FN‐coated wells (Figure 3A). Neither the addition of MBP‐scFv^K20^ nor its parent mAb K20 significantly affected cell attachment at 10 minutes (Figure 3A). The extent of cell adhesion after incubation for 30 minutes was similarly stimulated or inhibited in the presence of 9EG7 or AIIB2, respectively. In contrast, although there was a trend toward increased cell adhesion upon 30‐minute incubation with either MBP‐scFv^K20^ or mAb K20, their effects were not significant (Figure 3B).

Given this trend toward increased cell adhesion, we took a third approach to quantitatively measure the effects of MBP‐scFv^K20^ incubation on integrin function. H1975 cells were imaged after incubation for 30 minutes at 37°C in the absence (control) or presence of 5 μg/mL MBP‐scFv^K20^. Static immunofluorescence TIR‐FM images revealed no obvious effects, as the extent of cell attachment and spreading was variable under control and MBP‐scFv^K20^ incubation conditions (Figure 3C and Figure S2A). Integrin‐based cell‐ECM adhesions can induce integrin clustering and focal adhesion (FA) formation. As the turnover of adhesions is an essential part of integrin trafficking and is critical for efficient cell migration,^6^ we next examined whether MBP‐scFv^K20^ can affect focal adhesions. Quantitative analysis of TIR‐FM‐acquired immunofluorescence images revealed no significant effect of incubation with MBP‐scFv^K20^ for 30 minutes at 37°C on any of the measured parameters of FAs, including total FA area/cell area (Figure 3D), total FA area or the number of FA/cell area (Figure S2B,C, respectively). Taken together, we conclude that MBP‐scFv^K20^ binds specifically to β1 integrin, but does not significantly alter integrin or adhesion function.

### 2.3 Measuring β1 integrin uptake and recycling using MBP‐scFv^K20^

**FIGURE 4:**
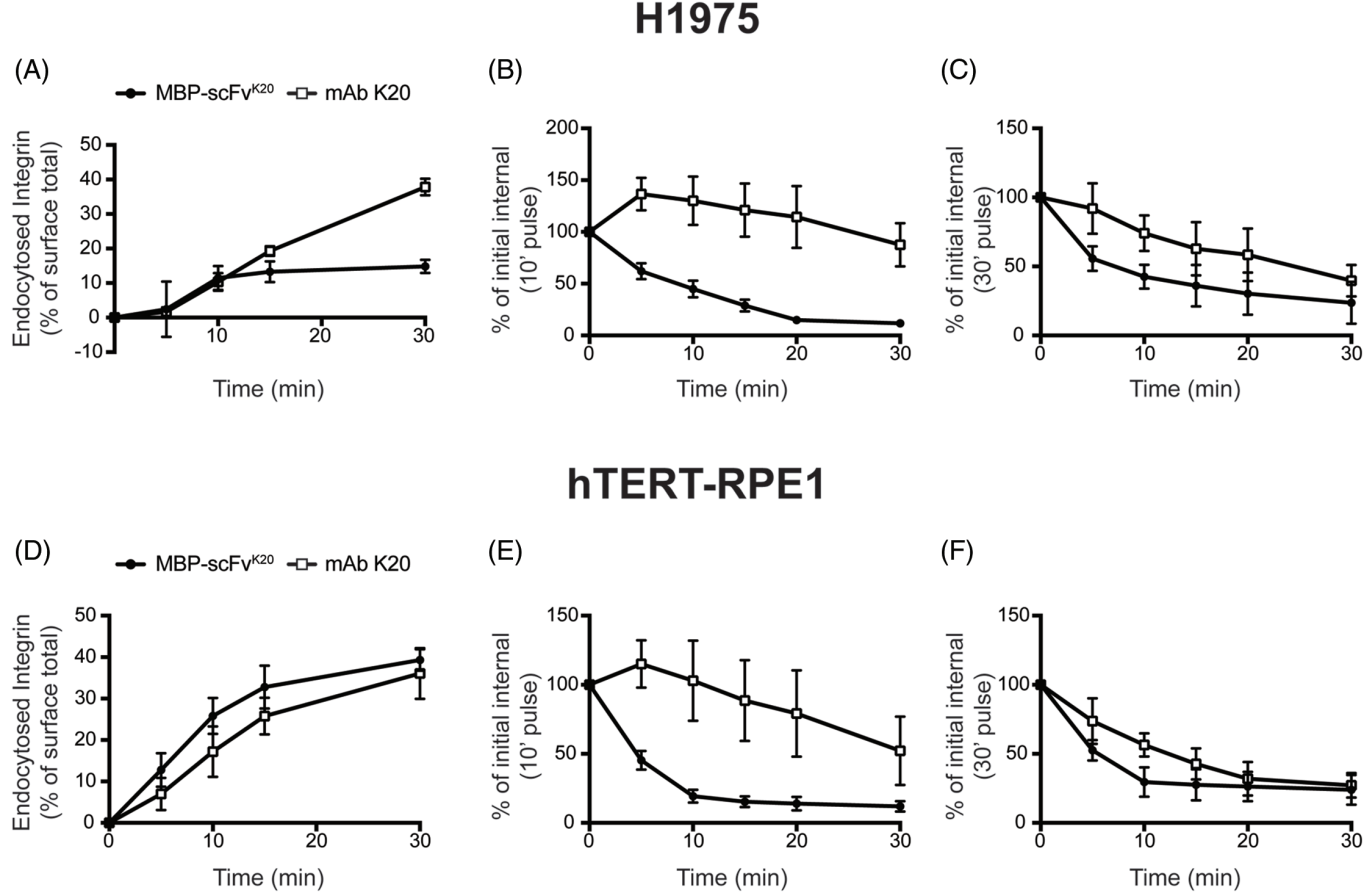
MBP‐scFv^K20^ serves as probe for biochemical assays of β1 integrin uptake and recycling. A, Endocytosis of biotinylated anti‐β1 integrin MBP‐scFv^K20^ (5 μg/mL) or mAb K20 IgG (1 μg/mL) in H1975 cells. Shown is the percentage of internalized antibody at the indicated times calculated relative to the initial surface bound at 4°C. B and C, Recycling of biotinylated‐MBP‐scFv^K20^ (5 μg/mL) or ‐mAb K20 IgG (1 μg/mL) in H1975 cells. Shown is the percentage of remaining intracellular biotinylated antibody at the indicated times relative to the initial internal loading of 10 minutes (B) or 30 minutes (C). All experiments represent n ≥ 3. Data plotted as mean ± SD. D‐F, As for A‐C except assays were performed in hTERT‐RPE1 cells

Our data indicated that MBP‐scFv^K20^ is functionally neutral and retains β1 integrin binding specificity. We next studied the utility of MBP‐scFv^K20^ as a biochemical probe to measure integrin uptake and recycling. The uptake of endogenous β1 integrin was measured by incubating H1975 cells in the continuous presence of biotinylated MBP‐scFv^K20^ or unlabeled mAb K20 for the indicated times at 37°C before returning cells to ice and stripping surface‐bound antibodies.^56^ Whereas mAb K20 continued to accumulate intracellularly in H1975 cells throughout the time course, intracellular MBP‐scFv^K20^ reached steady state after 15 minutes, suggestive of scFv recycling (Figure 4A).

The rapid recycling of integrins has been shown to play a role in cancer‐mediated cell migration; however, fast recycling kinetics (ie, after short internalization periods) have not been measured using previously available techniques, due to lack of sensitivity. Therefore, we next investigated whether MBP‐scFv^K20^ could be used to quantify the rapid recycling of β1 integrins, measured in the presence of 20 ng/mL EGF, which stimulates integrin trafficking.^20^, ^21^, ^33^ For this, both MBP‐scFv^K20^ and mAb K20 IgG were biotinylated via a cleavable disulfide bond. To independently measure both rapid and slow integrin recycling, cells were then subjected to either a short (10 minutes) or long (30 minutes) internalization pulse, respectively, at 37°C with either biotinylated‐mAb K20 or ‐MBP‐scFv^K20^. Cells were then immediately cooled to 4°C to stop internalization and washed with PBS containing TCEP to remove the biotin moiety on the remaining surface‐bound antibodies (see Section 4, Methods). For subsequent recycling measurements, cells were incubated at 37°C for the indicated times in the continuous presence of 20 ng/mL EGF and TCEP before returning cells to ice and stripping the cell surface‐bound antibodies. Our recycling assay measures the loss of internal signal over time, which, given the rapid kinetics most likely reflects receptor recycling to the plasma membrane rather than lysosomal degradation. Remaining intracellular biotin‐labeled antibodies were assessed and the percentage of recycling was calculated relative to the initial (10 or 30 minutes) internalization pulse. Biotinylated‐MBP‐scFv^K20^ exhibited efficient recycling in H1975 cells under conditions of fast (Figure 4B) and slow (Figure 4C) recycling, whereas the parent mAb K20 did not recycle after a 10‐minute internalization pulse (Figure 4B) and recycled with lower efficiency than MBP‐scFv^K20^ after the longer 30‐minute internalization pulse (Figure 4C).

We repeated these experiments in hTERT‐RPE1 cells and found that the internalization rates of both biotinylated‐mAb K20 and ‐MBP‐scFv^K20^ began to plateau after ~15 min (Figure 4D). Correspondingly, both probes exhibited significant recycling through fast and slow pathways (Figure 4E, F), although MBP‐scFv^K20^ recycled more rapidly and efficiently than mAb K20. We observed mAb K20 IgG signals above 100% at the 5‐minute time point in both cell lines during rapid integrin recycling assays (Figure 4B,E). This likely reflects incomplete dissociation of the bivalent mAb K20 relative to the monovalent MBP‐scFv^K20^ during the quick wash steps. Together, these data demonstrate that the nonperturbing and monovalent MBP‐scFv^K20^ can be used as a probe for biochemical β1 integrin uptake and recycling assays in multiple cell lines, and is superior to its parent mAb K20 for use in rapid integrin recycling assays.

### 2.4 Using scFv to track adhesions in cell‐based imaging assay

**FIGURE 5:**
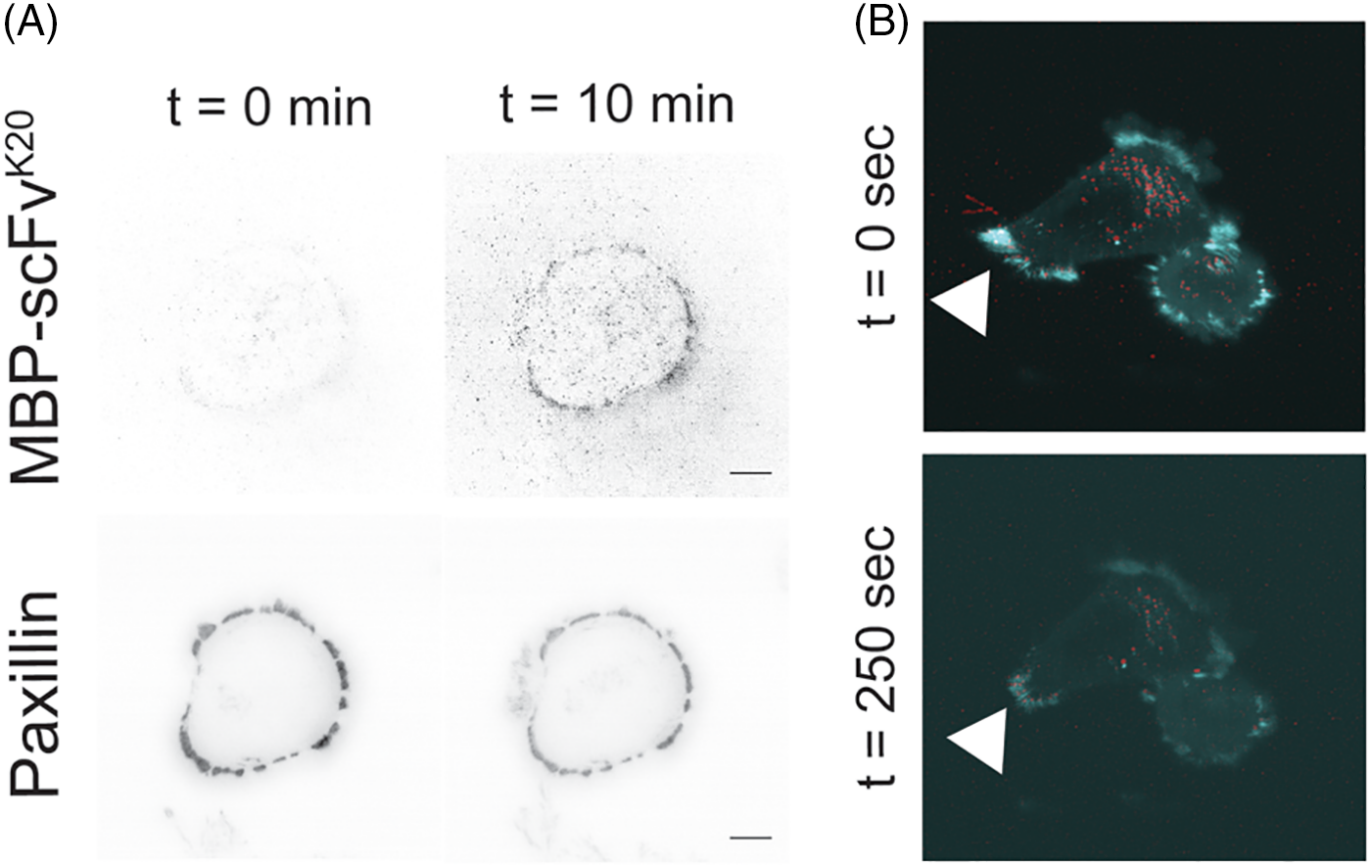
Anti‐β1 integrin MBP‐scFv^K20^ can track adhesions in live cells. A, Inverted static images from time‐lapse TIR‐FM or, B, Static blended XY maximum intensity projection (MIP) images from time‐lapse 3D LSFM (see Movie S1). H1975 cells expressing focal adhesion marker mRuby2‐Paxillin (B, cyan) were pulsed with 8 μg/mL Alexa Fluor 488‐conjugated MBP‐scFv^K20^ (B, red) and (A) immediately imaged by TIR‐FM or (B) after 30 minutes by LSFM. Images were acquired every 10 seconds for 10 minutes. A, Scale bar, 10 μm. B, White arrowheads indicate sites of adhesion disassembly

While the use of fluorescently labeled integrins does have drawbacks, it has enabled the imaging of integrins in live cells and provided initial insight into the spatiotemporal regulation of integrin trafficking. Given that MBP‐scFv^K20^ can track endogenous β1 integrin in biochemical assays, we next tested its ability to detect adhesion dynamics in live cells. To this end, H1975 cells stably expressing a fluorescently tagged adhesion reporter, mRuby2‐Paxillin, were seeded on gelatin‐ and FN‐coated coverslips, pulsed with an excess of Alexa Fluor‐conjugated MBP‐scFv^K20^ and imaged by live cell TIR‐FM (Figure 5A) or light sheet fluorescence microscopy (LSFM, Figure 5B and Movie S1). Static inverted TIR‐FM micrographs of cells imaged 10 minutes after addition of Alexa Fluor‐conjuated MBP‐scFv^K20^ revealed accumulation of MBP‐scFv^K20^ signal and colocalization of scFv with focal adhesion marker paxillin (Figure 5A). After longer incubation periods (≥30 minutes), cells imaged by 3D LSFM showed the association of fluorescent MBP‐scFv^K20^ (Figure 5B, red) with adhesion marker paxillin (cyan), as well as its accumulation in intracellular compartments (Figure 5B and Movie S1). We note that both live cell TIR‐FM and LSFM imaging displayed high background and low fluorescent MBP‐scFv^K20^ signals, culminating in rapid photobleaching. Nonetheless, these data establish the potential utility of scFv^K20^ for imaging integrin dynamics in living cells.

## 3 DISCUSSION

The field of integrin trafficking is rapidly expanding, largely because integrin function is vital for many fundamental cellular properties, such as cell identity, signaling, and motility. Therefore, new tools and assays to quantitatively track and measure endogenous integrins would help to further increase our understanding of integrin trafficking and its regulation. Here, we describe a functionally neutral and monovalent antibody‐based probe that can be used to study endogenous β1 integrin uptake and recycling in multiple cell types. We designed MBP‐scFv^K20^ to retain its β1 integrin‐specific binding capabilities while remaining monovalent by utilizing amino acid sequences obtained from the variable heavy and light antigen recognition domains of a widely used and non‐function perturbing murine anti‐β1 integrin monoclonal antibody, mAb K20. The direct comparison between the monovalent and bivalent antibody probes reveals significant advantages of MBP‐scFv^K20^ over mAb K20. Thus, MBP‐scFv^K20^ can help fill critical knowledge gaps by allowing for the biochemical characterization of rapid integrin recycling; however, it is still not a perfect tool. We discuss the limitations of scFv‐based technologies below, and offer MBP‐scFv^K20^ as an intellectual proof of principle for the future design and generation of nonperturbing and monovalent antibody‐based probes.

ScFvs are widely used in scientific research, as they are desirable for their small size, facile design, and can be expressed in several systems. We report high expression levels of our secreted scFv through baculovirus‐mediated insect cell expression. The benefits of this system are as follows: (1) it allowed for proper scFv disulfide bond formation, and (2) it facilitated easy purification by eliminating the need for cell lysis. While baculovirus generation can be difficult and time consuming,^57^ its use resulted in high levels of recombinant protein expression. The recent development of FlexiBAC, a versatile vector system for rapid baculovirus‐driven secreted protein production,^58^ has the potential to overcome burdensome time constraints of previous baculovirus expression systems.

While scFvs remain widely used, they are prone to aggregation and thus often require additional genetic tags to mitigate protein instability.^51^, ^54^ Although the addition of MBP increases both the solubility and stability of our scFv, it does effectively double the size of our probe. We therefore suggest that future integrin antibody‐based probe design limit increasing overall molecular size. Recent advances in monovalent camelid nanobody expression and purification suggest that generation of anti‐integrin nanobodies may greatly overcome the limitations inherent with current scFv‐based approaches.

MBP‐scFv^K20^ is designed to be versatile and customizable through differential labeling for each experimental condition. Although MBP‐scFv^K20^ is designed for in vitro site‐specific labeling by the protein SortaseA, there is a need for more commercially available reagents for “Sortagging.” While labeling MBP‐scFv^K20^ with commercially available amine‐reactive reagents is suitable for the assays performed herein, we do note that site‐specific labeling could reduce labeling‐induced heterogeneity and produce more consistent and reliable results by eliminating the batch‐to‐batch variability of different protein preparations. Additionally, to reduce unwanted aggregation, we used protein preparations with concentrations at or below 1 mg/mL and amine‐reactive crosslinkers at physiological pH, together resulting in low labeling efficiencies. In the case of biotinylation for the biochemical analysis of integrin internalization and recycling, labeling efficiencies were improved by employing a water‐soluble amine‐reactive NHS (*N*‐hydroxysuccinimide) ester, capable of reacting with primary amines at physiological pH. However, the most hydrolytically stable amine‐reactive moiety for direct Alexa Fluor dye conjugation uses a SDP (sulfodichlorophenol) ester, which requires more alkaline conditions (pH 8‐8.5) for optimized labeling. Low labeling efficiencies could be overcome by future steps taken to increase the expression and solubility of MBP‐scFv^K20^. Additionally, a genetically fused recombinant GFP‐scFv^K20^ could be used as an alternative to generate a fluorescently‐labeled scFv^K20^, given that GFP and MBP are similar in size and have demonstrated solubility enhancing properties.

Although MBP‐scFv^K20^ was designed for robust use in both biochemical and imaging‐based assays, we experienced issues with high background and poor MBP‐scFv^K20^ signals. As discussed, significant efforts were made to create a fluorescently‐labeled MBP‐scFv^K20^ for use in live cell imaging experiments. Live cell images were acquired with long exposure times (1 second) in 10‐second intervals to overcome the low SNR while simultaneously minimizing photobleaching. However, integrin trafficking is a dynamic process that is best studied by live fluorescence microscopy with high temporal resolution. Therefore, we are unable to reliably validate MBP‐scFv^K20^ as a tool to study the spatial regulation of β1 integrin without more robust labeling techniques.

We focused on designing an scFv to track endogenous β1 integrin because β1 integrin is expressed in nearly all cell types and can form heterodimers to recognize nearly every known ECM ligand. While this is advantageous in many regards, it is becoming increasingly appreciated that integrin heterodimers are under different regulatory mechanisms and can exhibit different trafficking itineraries.^10^, ^19^ We therefore propose that future antibody‐based probes be designed against specific αβ heterodimer pairs and/or designed in coordination for use with specific ECM ligands. The development of nanobody screening libraries^59^, ^60^ should aid in generating these heterodimer‐ and ECM‐specific reagents. Nonetheless, we believe the use of our tool will help to increase understanding of β1 integrin trafficking and its regulation in cells.

Finally, having established that MBP‐scFv^K20^ is functional, can be used to assess β1 integrin trafficking in biochemical assays, and associates with adhesions in live cells over time, we hope our analysis of MBP‐scFv^K20^ serves to inspire future integrin probe design. Specifically, we believe the generation of a single and monovalent antibody‐based tool, allowing for the correlation of bulk biochemical assays with spatiotemporal dynamics acquired through high‐resolution microscopy, will dramatically increase our knowledge of integrin trafficking and its roles in development and disease.

## 4 MATERIALS AND METHODS

### 4.1 Cell culture

H1975 cells were kindly provided by Dr. John Minna (UT Southwestern Medical Center at Dallas) and maintained at 5% CO_2_ at 37°C in biotin‐free RPMI 1640 medium (USBiological), supplemented with 10% (vol/vol) FCS (Fetal Calf Serum, Sigma). hTERT RPE1 cells were obtained from ATCC and maintained at 5% CO_2_ at 37°C in DMEM high‐glucose medium (Thermo Fisher Scientific) supplemented with 10% (vol/vol) FCS (Sigma). To reduce biotin background, all cells were maintained in media without added biotin. Sf9 (*Spodoptera frugiperda*, GIBCO‐BRL) insect cells were maintained at 27°C in Sf‐900 III SFM medium (GIBCO) supplemented with 2 mM l‐glutamine. High Five insect cells were generously provided by Dr. Vincent Tagliabracci (UT Southwestern Medical Center at Dallas) and maintained at 27°C in serum‐free ESF 921 medium (Expression Systems).

### 4.2 Generation and cloning of scFv

Amino acid sequences of the variable heavy (*V*_H_) and variable light (*V*_L_) chains from the mouse monoclonal anti‐β1 integrin antibody (mAb K20, Beckman Coulter) were obtained from Patent Ep0781337A1 “Humanised antibody to integrin chain β1” and are shown in Figure S1A. ScFv^K20^ was designed to have amino‐terminal *V*_H_ and carboxy‐terminal *V*_L_ sequences connected through a flexible linker (3× repeat of GGGGS). Additionally, a FLAG tag and SortaseA (SrtA) recognition motif were added to the carboxy terminus of the scFv sequence. A cDNA construct encoding scFv^K20^ amino acid sequence was codon optimized for mammalian expression and commercially synthesized (Genewiz, Inc.). To achieve high levels of secreted scFv^K20^ expression via insect cells, the synthesized gene product was further cloned into a modified pFastBacHT vector, pSMBP2, generously gifted by Dr. Vincent Tagliabracci (UT Southwestern Medical Center), via PCR amplification, digestion and ligation through the PCR‐generated flanking 5′ *BamHI* and 3′ *HindIII* restriction sites. PCR was performed using *PfuUltra II* Fusion HS DNA Polymerase (Agilent). All primers were synthesized by IDT (Integrated DNA Technologies), and all restriction enzymes and DNA ligases were obtained from New England Biolabs (NEB). K20‐scFv‐pSMBP2 is available on Addgene.

### 4.3 Bacmid and baculovirus generation

To generate bacmid DNA, K20‐scFv‐pSMBP2 plasmid was transformed into MAX Efficiency Chemically Competent DH10Bac *E. coli* cells (Life Technologies) following the recommended protocol. Briefly, DH10Bac competent cells were incubated with 1 ng of K20‐scFv‐pSMBP2 on ice. After a brief heat shock, the transformed competent cells were further incubated at 37°C for 4 hours to recover, and then plated on LB agar plates containing 50 μg/mL Kanamycin, 7 μg/mL gentamycin, 10 μg/mL tetracycline, 100 μg/mL Bluo‐gal, and 40 μg/mL IPTG and incubated at 37°C for 48 hours. White colonies were isolated, and re‐streaked on fresh plates. White colonies from the second round of plating were used for bacmid DNA isolation (Qiagen). Purified high molecular weight bacmid DNA was screened by PCR for proper gene transposition using pUC/M13 Forward (5′‐CCCAGTCACGACGTTGTAAAACG‐3′) and pUC/M13 Reverse (5′‐AGCGGATAACAATTTCACACAGG‐3′) primers (Life Technologies).

To generate recombinant baculovirus, Sf9 insect cells were transfected with bacmid DNA. Briefly, 8 × 10^5^ log‐phase suspension Sf9 cells were seeded in replicate wells of a 6‐well dish and allowed to adhere for 15 minutes at room temperature. Cells were transfected with 500 ng of recombinant bacmid DNA using Cellfectin II reagent (Life Technologies) according to the recommended protocol. After 4 hours, the transfection medium was removed and fresh Sf‐900 III SFM (GIBCO) medium containing antibiotics was added to cells. The cells were incubated without agitation at 27°C until signs of late‐stage viral infection were obvious (eg, signs of viral budding and cell lysis; approximately 5 days, and Figure S1B). The P1 viral supernatant was harvested and clarified and stored with 2% FCS final concentration at 4°C in the dark. To generate a high‐titer P2 baculovirus stock, the P1 viral supernatant was amplified by infecting 1.5 × 10^6^ cells/mL log‐phase Sf9 cells in suspension. P2 viral supernatant was collected after signs of late‐stage infection (approximately 4 days) and stored correspondingly.

### 4.4 Protein expression and purification

ScFv^K20^ was expressed by infecting 50 mL of log‐phase High Five insect cells at 1.5 × 10^6^ cells/mL in suspension with P2 recombinant baculovirus supernatant for 48 hours at 27°C. Clarified insect cell supernatant was collected and filtered through a 22 mm MCE 0.45 μm filter (Thermo Fisher Scientific) and kept on ice. Filtered supernatant containing the secreted recombinant scFv^K20^ was loaded directly into a pre‐chilled 50 mL superloop (GE Healthcare) and purified by FPLC (AKTÄ, GE Healthcare). Initial purification of scFv^K20^ was performed via immobilized metal ion affinity chromatography (IMAC) on a 1 mL HisTrap Excel column (GE Healthcare). The column was washed with 20 column volumes (CV) of Buffer A (20 mM sodium phosphate, 0.5 M NaCl, 20 mM imidazole pH 7.5), followed by a wash step to 25% Buffer B (20 mM sodium phosphate, 0.5 M NaCl, 250 mM imidazole pH 7.5) for 10 CV. Recombinant scFv^K20^ was eluted in two steps: a linear gradient to 100% Buffer B over 10 CV followed by 10 CV of 100% Buffer B. Fractions containing the MBP‐fused scFv after IMAC enrichment were pooled and concentrated using Amicon 10 kDa MWCO centrifugal spin filters (Millipore) before further purification and buffer exchange to PBS containing 5% glycerol (vol/vol) via FPLC size exclusion chromatography on a Superdex200Increase column (GE Healthcare). SEC resulted in two separate peaks containing the MBP‐fused scFv^K20^. Corresponding fractions from Peak 2 (Figure S1C) were pooled and concentrated using Amicon 10 kDa MWCO spin filters (Millipore). Final concentration of purified MBP‐scFv^K20^ was determined by bicinchoninic acid assay (BCA, Thermo Fisher Scientific) and used for subsequent labeling reactions.

### 4.5 Antibody labeling

Purified MBP‐scFv^K20^ was conjugated with a disulfide‐cleavable Biotin using EZ‐link NHS‐SS‐Biotin (Thermo Fisher Scientific) or Alexa Fluor 488 SPD (Invitrogen) according to manufacturer's guidelines. Briefly, purified MBP‐scFv^K20^ was incubated with 5‐fold molar excess NHS‐SS‐Biotin or Alexa Fluor 488 SPD at 4°C for 2 hours in PBS containing 5% glycerol (vol/vol) at pH 7.4. For biotinylation of mAb K20, antibody was incubated with 10‐fold molar excess NHS‐SS‐Biotin at 4°C for 2 hours in PBS at pH 7.4. Excess label was removed through a desalting step using Zeba spin desalt columns (Thermo Fisher Scientific). Single‐use aliquots of biotinylated‐ or Alexa Fluor‐conjugated MBP‐scFv^K20^, and biotinylated mAb K20 were snap frozen in LN_2_ and stored at −80°C in PBS containing 5% glycerol (vol/vol) or PBS, respectively. Freeze‐thaw cycles are not recommended.

### 4.6 Binding assay

H1975 cells (3.5 × 10^4^ cells/well) were seeded in biotin‐free RPMI for 6 hours on 0.02% gelatin‐ and fibronectin (25 μg/mL)‐coated 96‐well plates. At the time of the assay, cells were washed in cold PBS and incubated in complete biotin‐free medium for 10 minutes at 4°C. Cells were washed once more in cold PBS and incubated in the indicated antibody dilutions in PBS^4+^ buffer (PBS supplemented with 1 mM MgCl_2_, 1 mM CaCl_2_, 5 mM glucose and 1% bovine serum albumin) for 30 minutes at 4°C. Unbound antibody was removed by 3× PBS washes at 4°C. Cells were fixed in 4% PFA (Electron Microscopy Sciences) for 10 minutes at 4°C followed by 30 minutes at 37°C. Cells were washed with PBS twice and incubated in 1% casein/PBS overnight at 4°C to block nonspecific streptavidin binding. Surface‐bound MBP‐scFv^K20^ was assessed using streptavidin‐POD and surface‐bound mAb K20 was assessed using a goat anti‐mouse HRP‐conjugated antibody. The reaction was further developed with OPD (P1536, Sigma‐Aldrich), and then stopped by 5 M H_2_SO_4_. The absorbance was read at 490 nm (Biotek Synergy H1 Hybrid Reader). Well‐to‐well variability in cell number was normalized by a BCA assay (Thermo Fisher Scientific).

### 4.7 Adhesion and cell spreading assays

Adherent H1975 cells were brought into suspension by the addition of a 5 mM EDTA solution for 5 minutes at 37°C/5% CO_2_. Cells were collected and washed once with complete medium before further resuspension at 5 × 10^5^ cells/mL in complete medium. To test adhesion, 50 μL of cells were mixed with 50 μL of 2× antibody dilutions in complete medium (for a final antibody concentration of 5 μg/mL) and seeded in triplicate on black 0.02% gelatin‐ and fibronectin (25 μg/mL)‐coated 96‐well plates. To estimate attachment efficiency, a standard curve was generated by diluting cells 0%, 20%, 50% and 100% from the initial cell suspension in 50 μL of complete medium, which was added to wells containing 50 μL of complete medium. Cells were allowed to adhere for 10 or 30 minutes at 37°C/5% CO_2_ before 2× PBS washes. Remaining attached cells were fixed by adding 5% glutaraldehyde in PBS and incubated for 30 minutes at 37°C/5% CO_2_. Glutaraldehyde was removed with a 3× PBS wash step followed by a 60‐minute 0.1% crystal violet (wt/vol) staining step. Cells were washed 3× with PBS and the dye was solubilized by adding 10% (vol/vol) acetic acid for 5 minutes at room temperature. To determine cell attachment, the absorbance at 570 nm was read using a microplate reader (Biotek Synergy H1 Hybrid Reader). Background crystal violet staining (0% cells) was subtracted from all experimental conditions, and values are presented as normalized to the 100% adhesion control. Additional antibodies used in this assay are: mAb K20 (Beckman Coulter; neutral control), AIIB2 (Developmental Studies Hybridoma Bank, DSHB; inhibitory control), 9EG7 (BD Bioscience; activating control).

### 4.8 Endocytosis and recycling assays

Endocytic trafficking assays were performed using Corning Costar Stripwell 96‐well plates (Thermo Fisher Scientific). Plates were pre‐coated with 0.02% gelatin and 25 μg/mL human fibronectin (in PBS containing 2% sucrose) for 2 hours at 37°C then incubated in complete medium overnight at 4°C. hTERT RPE1 cells (2 × 10^4^ cells/well) were seeded overnight and H1975 cells (3.5 × 10^4^ cells/well) were seeded for 6 hours prior to experiments. To reduce biotin background, all cells were maintained in medium without added biotin.

For internalization experiments biotinylated MBP‐scFv^K20^ or unlabeled monoclonal β1 integrin antibody mAb K20 were used to track β integrin. We routinely use either bivalent anti‐transferrin receptor mAb D65 and disulfide‐cleavable biotinylated‐transferrin as ligand interchangeably to measure TfnR endocytosis and the kinetics of uptake are the same. Cells were incubated with 5 μg/mL biotinylated MBP‐scFv^K20^ or unlabeled 1 μg/mL mAb K20 in PBS^4+^ assay buffer at 37°C for the indicated time points and then immediately cooled to 4°C to stop internalization. The remaining surface‐bound biotinylated MBP‐scFv^K20^ or unlabeled mAb K20 was removed by an acid wash step (4× 30 seconds of 0.2 M acetic acid, 0.2 M NaCl pH 2.5). Cells were then washed 3× with cold PBS and fixed in 4% PFA (Electron Microscopy Sciences) in PBS for 30 minutes at 37°C. PFA was quenched with 100 mM glycine for 5 minutes at RT. Cells were permeabilized with 0.2% Triton X‐100 for 10 minutes at RT. Cells were then incubated in 1% casein/PBS overnight at 4°C to block nonspecific streptavidin binding. Internalized MBP‐scFv^K20^ was assessed using streptavidin‐POD and internalized mAb K20 was assessed using a goat anti‐mouse HRP‐conjugated antibody. The reaction was further developed with OPD, and then stopped by 5 M H_2_SO_4_. The absorbance was read at 490 nm (Biotek Synergy H1 Hybrid Reader). Well‐to‐well variability in cell number was normalized by a BCA assay (Thermo Fisher Scientific). Internalized ligand was expressed as the percentage of the total surface‐bound ligand at 4°C (ie, without acid wash step), measured in parallel.^56^

For recycling experiments both MBP‐scFv^K20^and mAb K20 were biotinylated with a cleavable disulfide bond to track β‐integrin. Cells were pulsed with biotinylated‐MBP‐scFv^K20^ or ‐mAb K20 and 20 ng/mL EGF in PBS^4+^ for 10 or 30 minutes at 37°C, to assess fast vs slow recycling, respectively. Cells were then immediately washed with PBS^4+^ and further incubated in PBS^4+^ containing 10 mM TCEP [tris(2‐carboxyethyl)phosphine] for 30 seconds at RT. TCEP reduces the disulfide bond releasing the biotin moiety. Cells were washed with cold PBS^4+^ and then incubated in PBS^4+^ containing 20 ng/mL EGF and 10 mM TCEP at 37°C for the indicated time points. Cells were then washed 4× in ice‐cold 0.2 M acetic acid/0.2 M NaCl (pH 2.5) followed by 3× cold PBS washes and fixed in 4% PFA (Electron Microscopy Sciences) in PBS for 30 minutes at 37°C and processed as above to determine the remaining intracellular biotinylated ligand. The decrease in intracellular biotinylated ligand (recycling) was calculated relative to the total pool of ligand internalized during the internalization pulse.

### 4.9 Immunofluorescence

Glass coverslips (22 × 22 mm) were coated with 0.01% poly‐l‐lysine for 10 minutes at RT, washed with PBS, then coated with 0.02% gelatin, and 25 μg/mL human fibronectin where indicated, in PBS containing 2% sucrose at 37°C for 15 minutes, followed by crosslinking with 1% PFA for 30 minutes at 37°C. Gelatin‐coated slips were extensively washed with PBS and incubated in complete medium at 4°C overnight. 2.5 × 10^5^ hTERT RPE1 or 3.5 × 10^5^ H1975 cells were seeded on gelatin‐ and fibronectin‐coated coverslips overnight. Cells were washed with PBS and incubated with antibody at the indicated concentrations for the indicated times before washing once with PBS, and fixation in 4% PFA for 30 minutes at 37°C. PFA was quenched in 100 mM glycine for 5 minutes at RT. Cells were washed with PBS, and permeabilized with 0.2% Triton X‐100 for 10 minutes at RT and then blocked in Q‐PBS (PBS containing 0.2% BSA, 0.001% saponin and 0.01% glycine) for 1 hour at RT. Fixed and permeabilized cells were then incubated in primary antibodies (1:500 dilution in Q‐PBS) at 4°C overnight. After three PBS washes, the cells were incubated with Alexa Fluor‐conjugated secondary antibodies or Streptavidin Alexa Fluor conjugate (1:1000 dilution in Q‐PBS) at 37°C for 1 hour. After three PBS washes, the cells were mounted in PBS and imaged by TIR‐FM. Primary antibodies used are: mouse anti‐β1 integrin mAb K20 (Beckman Coulter); mouseanti‐Paxillin (BD Biosciences); rabbit anti‐phospho‐Paxillin (Y118) (Cell Signaling Technologies).

### 4.10 Focal adhesion analysis

Focal adhesion analysis was performed using a previously published Focal Adhesion Analysis Package.^20^, ^61^, ^62^ Fixed‐cell paxillin immunofluorescence images were used to quantify total cellular adhesion density. The analysis software is available online at https://git.biohpc.swmed.edu/danuser/applications/pipelines/1944.

### 4.11 Total internal reflection fluorescence microscopy

Total internal reflection fluorescence microscopy (TIR‐FM) was performed as previously described.^63^ Briefly, cells were mounted in PBS and imaged using a 60×, 1.49 NA APO TIRF objective (Nikon) mounted on a fully motorized Nikon Ti‐Eclipse inverted microscope with Perfect Focus System and coupled to an Andor “Diskovery TIRF/Borealis widefield illuminator” equipped with an additional 1.8× tube lens (yielding a final magnification of ×108). TIR‐FM illumination was achieved using a Diskovery Platform (Andor Technology). For live cell experiments, cells were maintained at 37°C during imaging. Imaging sequences were acquired using a sCMOS camera with 6.5 μm pixel size (pco.edge).

### 4.12 Light sheet fluorescence microscopy

Light sheet fluorescence microscopy (LSFM) was performed as previously described.^64^ H1975 cells stably expressing mRuby2‐Paxillin were seeded for 6 hours at 37°C/5% CO_2_ on 5 mm round coverslips pre‐coated with 0.02% gelatin and 25 μg/mL FN. Cells were mounted in a custom sample holder for imaging.^64^ Images were acquired every 10 seconds for 10 minutes.

## CONFLICT OF INTEREST

The authors declare no conflicts of interest.

## AUTHOR CONTRIBUTIONS

Ashley M. Lakoduk designed the project and carried out the study. Zuzana Kadlecova helped in the patent search and initial scFv design and performed immunofluoresence in Fig. 2C. Ashley M. Lakoduk and Sandra L. Schmid discussed and analyzed the results and wrote the manuscript.

## Supporting information


**Figure S1**: Validation of anti‐β1 integrin scFv^K20^ design, baculovirus expression, and purification. A, Amino acid sequences of the variable heavy (*V*_H_) and variable light (*V*_L_) domains of the recombinant scFv^K20^ and its parent murine monoclonal antibody, mAb K20. Residues in red represent amino acids identified by mass spectrometry analysis of peptides derived from purified Fab fragments of commercially‐available mAb K20 (Beckman Coulter). B, Bright field image of Sf9 insect cells 5 days post‐transfection with bacmid DNA. Cells exhibit signs of late‐stage viral infection (eg, viral budding and cell lysis). C, Representative FPLC size exclusion chromatogram of recombinant MBP‐scFv^K20^ purification. Blue peaks indicate relative protein abundance (Y‐axis: relative protein abundance measured by A_280_, mAU). Pink dashed line represents sample injection and brown line represents measured solution conductivity throughout the FPLC run (*X* axis: elution volume in mL and corresponding fractions in red). Peak 1 represents aggregated protein eluted in the column void volume. MBP‐scFv^K20^ eluting in peak 2 (fractions 22‐25, marked with an asterisk), was harvested and used for all experiments.
**Figure S2**: ScFv^K20^ does not perturb integrin function. A, Quantitative comparison of total detected focal adhesion (FA) area, and B, total detected FA density of (C) inverted TIR‐FM immunofluorescence images of H1975 cells seeded on gelatin‐ and fibronectin‐coated coverslips incubated in the absence (control) or presence of 5 μg/mL MBP‐scFv^K20^ for 30 minutes at 37°C. Scale bar, 10 μm. n.s., not significant. Wilcoxon Rank‐Sum non‐parametric test was used for statistical significance.

**Movie S1**: Anti‐β1 integrin MBP‐scFv^K20^ can track adhesions in live cells. H1975 cells expressing focal adhesion marker mRuby2‐Paxillin (cyan) were seeded on gelatin‐ and FN‐coated coverslips and pulsed with 8 μg/mL Alexa Fluor 488‐conjugated MBP‐scFv^K20^ (red) for ≥30 minutes and imaged by LSFM. Images were acquired every 10 seconds for 10 minutes. Dual‐color time lapse XY maximum intensity projection (MIP) are accompanied by non‐isotropic XZ (bottom) and YZ (right) MIP.
