# Supplementary material for: A functionally neutral single chain antibody to measure beta‐1 integrin uptake and recycling
Source: Traffic. 2020 Jul 21;21(9):590–602. doi: 10.1111/tra.12754 (PMC7442622; doi:10.1111/tra.12754)
Supplement: Supplementary file 1 — Figure S1: Validation of anti‐β1 integrin scFvK20 design, baculovirus expression, and purification. A, Amino acid sequences of the variable heavy (V H) and variable light (V L) domains of the recombinant scFvK20 and its parent murine monoclonal antibody, mAb K20. Residues in red represent amino acids identified by mass spectrometry analysis of peptides derived from purified Fab fragments of commercially‐available mAb K20 (Beckman Coulter). B, Bright field image of Sf9 insect cells 5 days post‐transfection with bacmid DNA. Cells exhibit signs of late‐stage viral infection (eg, viral budding and cell lysis). C, Representative FPLC size exclusion chromatogram of recombinant MBP‐scFvK20 purification. Blue peaks indicate relative protein abundance (Y‐axis: relative protein abundance measured by A280, mAU). Pink dashed line represents sample injection and brown line represents measured solution conductivity throughout the FPLC run (X axis: elution volume in mL and corresponding fractions in red). Peak 1 represents aggregated protein eluted in the column void volume. MBP‐scFvK20 eluting in peak 2 (fractions 22‐25, marked with an asterisk), was harvested and used for all experiments. Figure S2: ScFvK20 does not perturb integrin function. A, Quantitative comparison of total detected focal adhesion (FA) area, and B, total detected FA density of (C) inverted TIR‐FM immunofluorescence images of H1975 cells seeded on gelatin‐ and fibronectin‐coated coverslips incubated in the absence (control) or presence of 5 μg/mL MBP‐scFvK20 for 30 minutes at 37°C. Scale bar, 10 μm. n.s., not significant. Wilcoxon Rank‐Sum non‐parametric test was used for statistical significance. [file TRA-21-590-s001.docx]

**A functionally neutral single chain antibody to measure beta-1 integrin uptake and recycling**

Ashley M. Lakoduk^1^, Sandra L. Schmid^1,2^

^1^Department of Cell Biology,

UT Southwestern Medical Center, Dallas, TX 75390

^2^Lead Contact

Correspondence: Sandra L. Schmid ([Sandra.schmid@UTSouthwestern.edu)](mailto:Sandra.schmid@UTSouthwestern.edu)); Ashley M. Lakoduk ([Ashley.Lakoduk@UTSouthwestern.edu](mailto:Ashley.Lakoduk@UTSouthwestern.edu))

**Supplemental Material**

**Supplemental Movie S1.** **Anti-β1 integrin MBP-scFv^K20^ can track adhesions in live cells**

H1975 cells expressing focal adhesion marker mRuby2-Paxillin (cyan) were seeded on gelatin- and FN-coated coverslips and pulsed with 8 μg/ml Alexa Fluor 488-conjugated MBP-scFv^K20^ (red) for ≥ 30 minutes and imaged by LSFM. Images were acquired every 10 seconds for 10 minutes. Dual-color time lapse XY maximum intensity projection (MIP) are accompanied by non-isotropic XZ (bottom) and YZ (right) MIP.


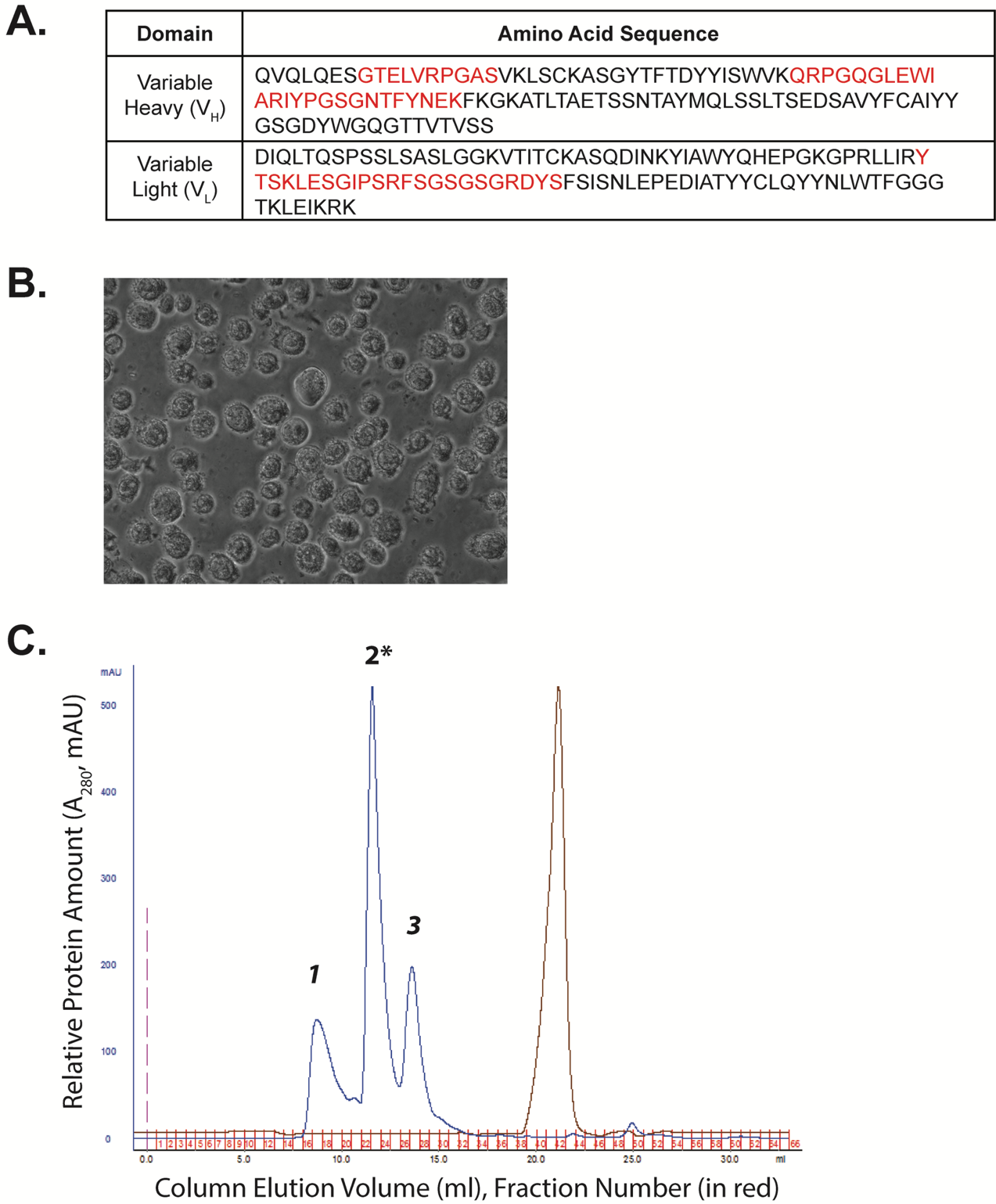


**Supplemental Figure 1. Validation of anti-β1 integrin scFv^K20^ design, baculovirus expression, and purification**.

(A) Amino acid sequences of the variable heavy (V_H_) and variable light (V_L_) domains of the recombinant scFv^K20^ and its parent murine monoclonal antibody, mAb K20. Residues in red represent amino acids identified by mass spectrometry analysis of peptides derived from purified Fab fragments of commercially-available mAb K20 (Beckman Coulter). (B) Bright field image of Sf9 insect cells five days post-transfection with bacmid DNA. Cells exhibit signs of late-stage viral infection (e.g. viral budding and cell lysis). (C) Representative FPLC size exclusion chromatogram of recombinant MBP-scFv^K20^ purification. Blue peaks indicate relative protein abundance (Y-axis: relative protein abundance measured by A_280_, mAU). Pink dashed line represents sample injection and brown line represents measured solution conductivity throughout the FPLC run (X axis: elution volume in ml and corresponding fractions in red). Peak 1 represents aggregated protein eluted in the column void volume. MBP-scFv^K20^ eluting in peak 2 (fractions 22-25, marked with an asterisk), was harvested and used for all experiments.


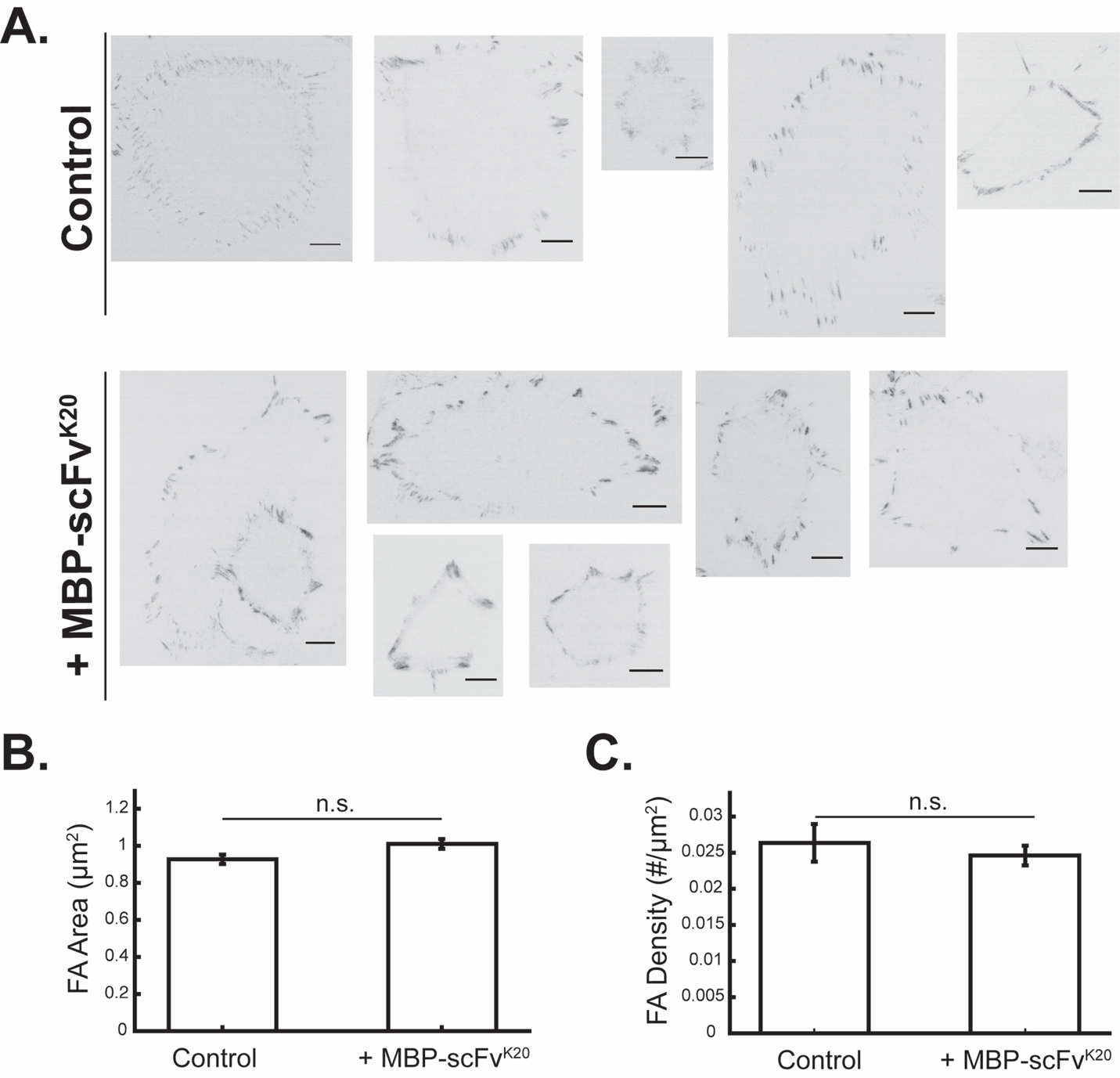


**Supplemental Figure 2. ScFv^K20^ does not perturb integrin function.**

(A) Quantitative comparison of total detected focal adhesion (FA) area and (B) total detected FA density of (C) inverted TIR-FM immunofluorescence images of H1975 cells seeded on gelatin- and fibronectin-coated coverslips incubated in the absence (control) or presence of 5 μg/ml MBP-scFv^K20^ for 30 minutes at 37°C. Scale bar, 10 μm. n.s.= not significant. Wilcoxon Rank-Sum non-parametric test was used for statistical significance.
